# Quartet metabolite reference materials for inter-laboratory proficiency test and data integration of metabolomics profiling

**DOI:** 10.1186/s13059-024-03168-z

**Published:** 2024-01-24

**Authors:** Naixin Zhang, Qiaochu Chen, Peipei Zhang, Kejun Zhou, Yaqing Liu, Haiyan Wang, Shumeng Duan, Yongming Xie, Wenxiang Yu, Ziqing Kong, Luyao Ren, Wanwan Hou, Jingcheng Yang, Xiaoyun Gong, Lianhua Dong, Xiang Fang, Leming Shi, Ying Yu, Yuanting Zheng

**Affiliations:** 1 State Key Laboratory of Genetic Engineering, School of Life Sciences and Human Phenome Institute, Shanghai Cancer Center, Fudan University, Shanghai, China; 2 Human Metabolomics Institute, Inc., Shenzhen, Guangdong China; 3 Shanghai Applied Protein Technology Co. Ltd, Shanghai, China; 4 Novogene Bioinformatics Institute, Beijing, China; 5 Calibra Diagnostics, Hangzhou, Zhejiang China; 6 Greater Bay Area Institute of Precision Medicine, Guangzhou, Guangdong China; 7 National Institute of Metrology, Beijing, China; 8 International Human Phenome Institute, Shanghai, China

**Keywords:** Metabolomics, Reference material, Quality control, Signal-to-noise ratio (SNR), Standardization, LC–MS

## Abstract

**Background:**

Various laboratory-developed metabolomic methods lead to big challenges in inter-laboratory comparability and effective integration of diverse datasets.

**Results:**

As part of the Quartet Project, we establish a publicly available suite of four metabolite reference materials derived from B lymphoblastoid cell lines from a family of parents and monozygotic twin daughters. We generate comprehensive LC–MS-based metabolomic data from the Quartet reference materials using targeted and untargeted strategies in different laboratories. The Quartet multi-sample-based signal-to-noise ratio enables objective assessment of the reliability of intra-batch and cross-batch metabolomics profiling in detecting intrinsic biological differences among the four groups of samples. Significant variations in the reliability of the metabolomics profiling are identified across laboratories. Importantly, ratio-based metabolomics profiling, by scaling the absolute values of a study sample relative to those of a common reference sample, enables cross-laboratory quantitative data integration. Thus, we construct the ratio-based high-confidence reference datasets between two reference samples, providing “ground truth” for inter-laboratory accuracy assessment, which enables objective evaluation of quantitative metabolomics profiling using various instruments and protocols.

**Conclusions:**

Our study provides the community with rich resources and best practices for inter-laboratory proficiency tests and data integration, ensuring reliability of large-scale and longitudinal metabolomic studies.

**Supplementary Information:**

The online version contains supplementary material available at 10.1186/s13059-024-03168-z.

## Background

Metabolomics is a powerful tool for discovering biomarkers that discriminate biological differences in metabolite abundances associated with disease diagnosis, prognosis, and treatment effects [1, 2]. However, reliably detecting such subtle biological differences is challenging due to technical variations introduced by various instruments and laboratory-developed protocols [3–7]. Moreover, in large metabolomics cohort studies, batch effects are inevitable when integrating datasets from multiple batches across laboratories and over long-term measurements [5, 8–10]. Thus, it is crucial to assure the reliability of each batch of metabolomic measurement as well as the integration of multiple batches of data in long-term or cross-laboratory studies so that the real signals (biological differences) can be distinguished from technical noises (unwanted variations) [11–14].

Publicly available reference materials (RMs) are indispensable for performance assessment in current practices [15–21]. At present, metabolite RMs are primarily developed and distributed by the U.S. National Institute of Standards and Technology (NIST), covering many biospecimen types such as plasma, serum, urine, and liver [22–24]. These various types of RMs and corresponding reference datasets enable the assessment of metabolomics profiling performance in different senarios [25, 26]. However, there is a lack of renewable metabolite reference materials from cultured cell lines, a crucial sample type in metabolomics studies.

Quality control (QC) metrics for objective performance evaluation are critically important. Reproducibility is one of the most widely used QC metrics, as exemplified by correlations or coefficients of variation [27, 28]. It helps assess the level of unwanted variations introduced by the sample processing and detection procedures through repeated measurements of a common reference sample [29]. However, high reproducibility from repeated measurements of the same sample does not guarantee high resolution in identifying inherent biological differences (i.e., signals) among various sample groups. Identification of differentially expressed metabolites and development of predictive models to classify different sample groups are the two major goals of quantitative metabolomics profiling. Therefore, QC metrics pertinent to such research purposes are crucial to assessing the performance of metabolomics profiling [30, 31]. Accuracy is another important QC metric, which is assessed through comparison of the measured metabolite concentrations with the “ground truth” in the reference datasets [25]. However, to the best of our knowledge, it is unachievable to define untargeted metabolomic reference datasets, wherein the quantitatively measured values are usually calculated as the relative output of instrumental response, which is notoriously incomparable between batches, protocols, instruments, or laboratories. To ensure the accurate identification of biological differences in discovering clinical biomarkers, accuracy assessment of untargeted metabolomics quantification is essential. Therefore, the development of quality metrics and best practices for proficiency testing of a wide range of metabolomic technologies is urgently needed [32].

Reliable integration of large-scale metabolomic data is a prerequisite for robust biomarker discovery and validation. Even if the intra-batch data is of high quality, batch effects are everywhere in large-scale metabolomics studies. In-house QC samples are widely used for long-term measurement within a single laboratory. Profiling QC samples along with study samples helps to assess the stability of measurement in each batch and to ensure efficient integration of multiple batches by removing batch effects introduced by unwanted variations over a time span [27, 33–38]. A pooled QC sample in the form of a mixture of the study samples has been widely used in this scenario, but it failed to ensure reliable data integration, mainly because the “pooled QC sample” is not identical across studies or across laboratories [35, 39, 40]. As a result, the lack of reliable data integration solutions hampers long-term, cross-laboratory, and cross-study exploration of new biological insights [32].

As part of the Quartet Project (chinese-quartet.org) for the quality control and data integration of multiomics profiling, we established the publicly available Quartet metabolite RMs and reference datasets. The Quartet metabolite RMs enabled the research purpose related QC metric, i.e., the multi-sample-based signal-to-noise ratio (SNR), for assessing the ability to discriminate the inherent biological differences among sample groups. In addition, we also demonstrated that ratio-based metabolomics profiling using common reference materials can enable long-term and cross-laboratory data integration in large-scale and multi-center metabolomic studies.

## Results

### Overview of the study design

**Fig. 1:**
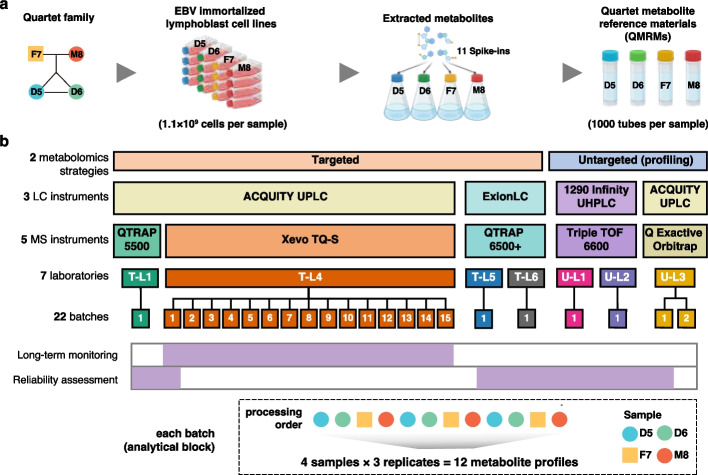
Study overview. **a** Preparation of the Quartet metabolite reference materials. Four B lymphoblastoid cell lines (LCLs) of a family quartet including the father (F7), mother (M8), and monozygotic twin daughters (D5 and D6) were used for extracting metabolites. Eleven spike-ins were added to the cell extract and aliquoted into 1000 tubes per sample. **b** Data generation. Targeted (T) and untargeted (U)-based metabolomic datasets were generated in different laboratories for inter-laboratory proficiency tests. Long-term monitoring was conducted using a targeted strategy within a laboratory (T-L4) for 2 years

In this study, we aim to provide the community with a suite of metabolite reference materials (RMs) and reference datasets for the inter-laboratory proficiency test and integration. The Quartet metabolite RMs were prepared as part of the Quartet Project, in which matched reference materials of DNA, RNA, proteins, and metabolites were simultaneously manufactured from the same batch of cultured cells. Four immortalized B lymphoblastoid cell lines were derived from a Chinese quartet family, including the father (F7), mother (M8), and their monozygotic twin daughters (D5 and D6) (Fig. 1a). In order to make the reference materials homogeneous and stable for long-term usage, a large batch of cell pellets (10^9^ cells per cell line) were extracted simultaneously using a methanol to water (6:1) solution (Additional file 1: Fig. S1). Eleven external controls were then added to the cellular extracts at known amounts (Additional file 2: Table S1). The cellular extracts were aliquoted into 1000 vials per cell line and then vacuum frozen and dried. Each vial of the Quartet metabolite RM contains dried cellular metabolites extracted from approximately 10^6^ cells, which are suitable for most liquid chromatography and mass spectrometry (LC–MS)-based metabolomics profiling. Additionally, the metabolite RMs were formulated as dried cellular extracts, so they are not suitable for assessing the pre-analytical steps such as cell extraction, but they are intended to evaluate the performances of chromatography separation, mass spectrometry detection, and data processing steps.

For the inter-laboratory proficiency test of metabolomics profiling, we generated multi-laboratory datasets using untargeted and targeted strategies (Fig. 1b). Three replicates of each Quartet sample were measured within a batch in each laboratory. Each metabolomic profile has been developed independently using different LC-MS experimental methods as well as different data processing strategies (Additional file 2: Table S2). Quantification for the targeted metabolomics was mostly carried out by relative metabolite abundance detected by multiple reaction monitoring (MRM), while one laboratory (T-L4) used an approach to calculate the metabolite concentration with standard calibration curves. Quantification for the untargeted metabolomic techniques was carried out using the relative metabolite abundances measured by precursor ions. For long-term stability monitoring of the Quartet metabolite RMs, three replicates of each Quartet sample were measured  every 1-3 months in T-L4 for 2 years.

### High variabilities in the intra-laboratory performance of metabolomics profiling

**Fig. 2:**
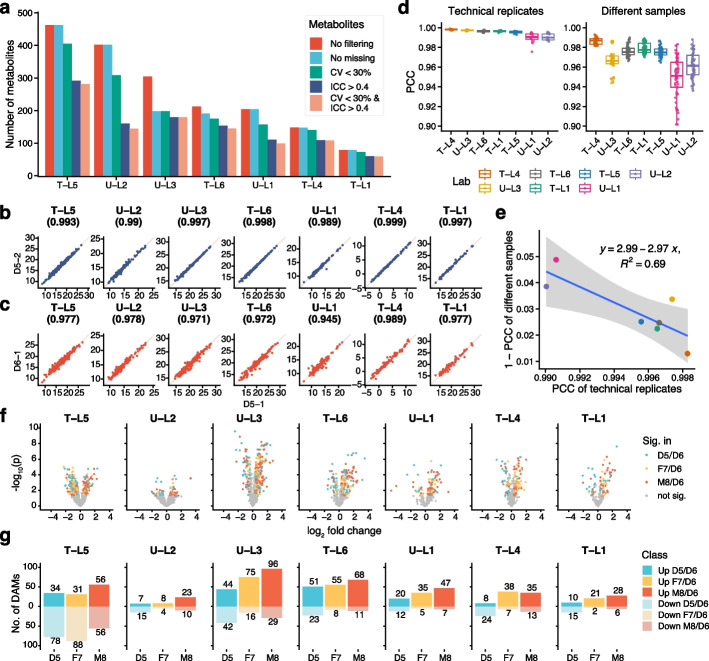
High variabilities in the performance of metabolomics profiling among laboratories. **a** Numbers of detected metabolites in each metabolomic measurement using different filtering criteria, including no filtering (all detected metabolites in any of the samples), no missing (metabolites detected in all 12 samples); CV (coefficient of variance) < 30%; ICC (intraclass correlation coefficient) > 0.04; CV < 30% and ICC > 0.04. **b** Reprehensive scatter plots of technical replicates (D5-1 and D5-2). **c** Reprehensive scatter plots of different samples (D5-1 and D6-1). Number in parentheses represent corresponding Pearson correlation coefficient (PCC). **d** PCC of pairs of technical replicates and of different Quartet samples in each measurement. **e** Negative correlation between reproducibility (PCC of technical replicates) and discriminability (1-PCC of different samples). **f** Differentially abundant metabolites (DAMs) analysis for three sample pairs. Volcano plots were used to display the magnitude of the fold change versus the statistical significance level for each measurement. **g** Numbers of upregulated or downregulated DAMs identified for three sample pairs in each measurement

We first evaluated the qualitative and quantitative performance of each metabolomic profile using different instruments and protocols. The number of metabolites detected by each laboratory varied from 79 to 462 (Fig. 2a). Untargeted metabolomic strategies are usually regarded as a tool for profiling all metabolites present in a sample. However, there was no obvious advantage in the number of detected metabolites using untargeted strategies. For example, only 204 metabolites were detected in untargeted metabolomics profiling in U-L1, whereas 463 metabolites were detected in targeted metabolomics in T-L5. We also compared the number of detected metabolites using different filtering criteria. The coefficient of variance (CV) was used to evaluate the reproducibility of technical replicates of the same sample, whereas the intraclass correlation coefficient (ICC) was a widely used reliability index in test–retest scenarios. After filtration with combined criteria of CV < 30% and ICC > 0.4, the percentages of detected metabolites ranged from 36 to 90%. A total of 402 metabolites were detected in U-L2 using an untargeted strategy, but only 36% of them were retained after filtration (Fig. 2a). On the other side, 304 metabolites were detected in U-L3, and 59% of them were retained after filtration (Fig. 2a).

The reproducibility of quantitative profiles was evaluated by the Pearson correlation coefficient (PCC) of pairs of technical replicates, while the similarity was evaluated by the PCC of pairs of different Quartet metabolite RMs. As shown in Fig. 2b, the PCC of technical replicates of D5 was high in all seven datasets, ranging from 0.989 to 0.999. However, the PCC between D5 and D6 was also high, ranging from 0.945 to 0.989 (Fig. 2c). For example, the PCC of technical replicates of D5 in T-L4 was 0.999, while the PCC between D5 and D6 in the same laboratory was 0.989. It implicated that high reproducibility of technical replicates and high similarity between different sample groups were usually concurrent in the same measurement (Fig. 2d). In addition, there was a negative correlation between the PCC of technical replicates and the 1-PCC of different sample groups (Fig. 2e). These results demonstrated that high reproducibility of technical replicates does not guarantee high resolution in identifying inherent biological differences (discriminability) between different sample groups.

One of the main objectives of metabolomic studies utilizing various protocols is the identification of differentially abundant metabolites (DAMs) for potential biomarker discovery. However, when comparing the DAMs between the same sample pairs, we observed large differences in both the fold changes and statistical significance levels (Fig. 2f). The number of DAMs ranged from 12 to 125 in D5/D6, F7/D6, and M8/D6 comparisons (Fig. 2g). Two of the laboratories (T-L5 and U-L3) identified higher numbers of DAMs, though the differential abundance patterns varied. Most of the metabolites were more abundant in D6 than in D5, F7, and M8 at T-L5, but the pattern was reversed at U-L3. These results revealed that high variabilities were observed in DAMs among different laboratories; hence, it cannot be determined which profiling method is of better quality.

### Quartet-based signal-to-noise ratio enables intra-laboratory reliability assessment

**Fig. 3:**
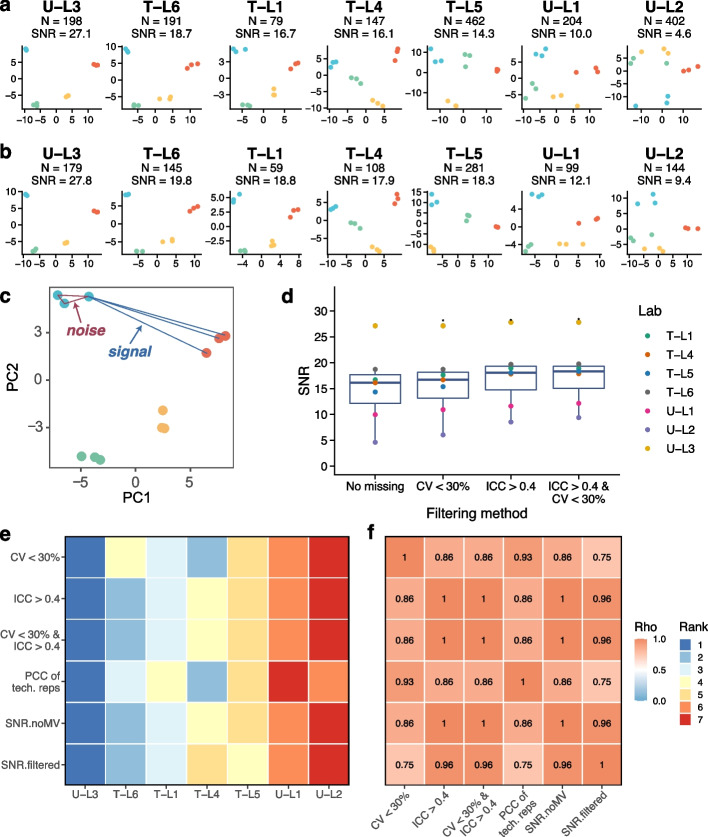
Quartet-based signal-to-noise ratio enables intra-laboratory reliability assessment. **a**, **b** Reliability assessment using signal-to-noise ratio (SNR) in different laboratories with (**a**) or without (**b**) filtration. The results were visualized by PCA plots. The number of features used and the calculated SNR were shown above each plot. **c** Schematic diagram of SNR calculated as the ratio of the averaged distance between different Quartet samples (“signal”) to the averaged distances between technical replicates for each sample (“noise”) on a 2D-PCA scatter plot. **d** SNRs calculated with metabolites filtered with different criteria across all laboratories. **e** The inter-laboratory data quality was ranked using different QC metrics, including the percentages of retained metabolites using different filtering criteria (CV < 30%; ICC > 0.4; CV < 30% and ICC > 0.4), PCC of technical replicates, and SNR calculated with or without filtering. **f** The concordances (Spearman Rho) of data quality ranking using different QC metrics

Based on the Quartet multi-sample design, we designed a signal-to-noise ratio (SNR) metric to measure the ability of metabolomics profiling to discriminate biological differences among different sample groups. SNR is calculated as the ratio of the averaged distance between different Quartet samples (“signal”) to the averaged distances between technical replicates for each sample (“noise”) on a 2D-PCA scatter plot (Fig. 3c) [41, 42], where a higher SNR indicates better quality. As expected, for a measurement to be considered reliable, the sample-to-sample differences should be larger than the variation of technical replicates.

We computed the SNR for each batch of metabolomics profiling (4 samples × 3 replicates) using metabolites detected in all 12 samples within the batch. As shown in the PCA, the first two principal components demonstrated clear separation among the four reference samples in good-quality metabolomics profiling data but not in poor-quality data (Fig. 3a). Astonishingly, high variabilities in data quality were observed in these metabolomic datasets (range of 4.6–27.1). After filtering (CV < 30% and ICC > 0.4) to retain the reliably detectable metabolites, the SNRs were slightly improved, but the relative quality ranking of batches did not change (Fig. 3b). Figure 3d illustrates the SNRs calculated with metabolites filtered with different criteria, indicating the robustness of SNRs in evaluating laboratory-specific reliability with or without filtration.

We also ranked the quality of the metabolomics profiling datasets generated in different laboratories using various QC metrics, including the percentages of retained metabolites using different filtering criteria (CV < 30%; ICC > 0.4; CV < 30% and ICC > 0.4), PCC of technical replicates, and SNR calculated with or without filtering. As shown in Fig. 3e, the inter-laboratory data quality rankings were not entirely concordant using different QC metrics. T-L4 performed well by PCC of technical replicates but did not perform well by SNR. The overall concordances among these QC results were also evaluated in Fig. 3f. These results suggested that the correlation of replicates from one reference material did not have enough resolution power to identify the multi-sample differences. The Quartet multi-sample-based SNR provided an objective QC metric for inter-laboratory reliability assessment for a wide range of metabolomic technologies.

### Ratio-based metabolomics profiling enables quantitative data integration across laboratories

**Fig. 4:**
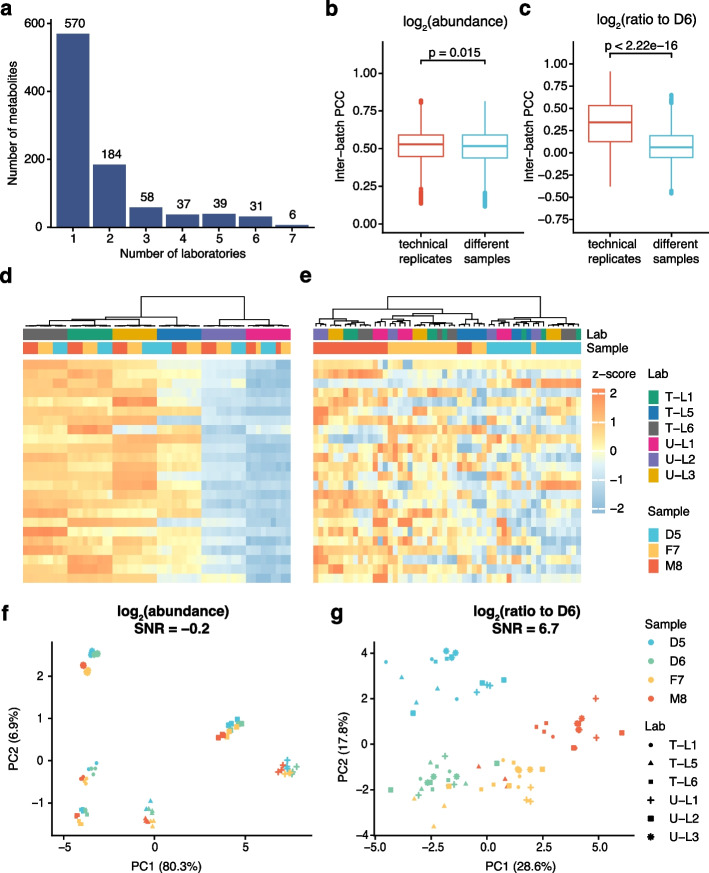
Ratio-based metabolomics profiling enables quantitative data integration across laboratories. **a** Qualitative concordance of metabolite identification. The numbers of metabolites detected in different batches of metabolomic datasets were shown. **b**, **c** Pearson correlation coefficients (PCCs) of pairs of technical replicates (**b**) and of different Quartet samples (**c**) were compared using quantitative profiles at absolute abundance level or ratio to D6 level. **d**, **e** Cross-lab data integration was visualized by hierarchical cluster analysis (HCA) at the absolute abundance level (**d**) and ratio to D6 level (**e**). **f**, **g** Cross-lab data integration assessment using signal-to-noise ratio (SNR) by principal component analysis (PCA) at absolute abundance level (**f**) and ratio to D6 level (**g**)

To evaluate the reliability of metabolomic data integration, we examined the qualitative and quantitative performance of the integrated data generated in different laboratories. We first evaluated the qualitative concordance of detected metabolites among these datasets. Only six metabolites were reported in each of the seven metabolomic datasets, and the majority of detected metabolites were reported by just one laboratory (Fig. 4a). The intersection size of detected metabolites among different laboratories is shown in Additional file 1: Fig. S2, and there were only 58 metabolites detected by all three global untargeted metabolomics profiling. Selectivity bias of detected metabolites was expected because the laboratory-developed metabolomics profiling approaches use different chromatography separation, mass spectrometry detection, data processing, and metabolite identification methods. However, our results objectively revealed the poor concordance of metabolite detections across different laboratories using common reference materials.

To further evaluate the quantitative reliability of integrating six batches of metabolomic datasets at the absolute abundance level, we first compared the differences between PCCs of technical replicates and those between different sample groups. However, the differences between the two types of PCCs were not significant (Fig. 4b). Hierarchical cluster analysis (HCA) and principal component analysis (PCA) were also used to visualize the magnitude of technical variation in data integration at the absolute abundance level. The integrated metabolomic data were first clustered by laboratory but not by different sample groups (Fig. 4d). Similar results were demonstrated in PCA (Fig. 4f), where the first principal component (PC1) clearly showed the dramatic differences between the six batches of data but not between the distinct Quartet samples.

Importantly, significant differences between PCCs of technical replicates and those of different samples were discovered after converting the absolute abundance data to a ratio scale relative to the same reference material (Here, D6) in each batch (Fig. 4c). HCA and PCA plots showed similar results (Fig. 4e, g). After the ratio-based scaling, the metabolomics profiling relative to D6 first clustered by the four different Quartet sample groups. PCA plots showed clear separation of the four groups of reference samples (D5, D6, F7, and M8), and the drastic batch effects at the absolute abundance level largely disappeared. Our results showed that batch effects were prevalent in cross-laboratory metabolomic data integration at the absolute abundance level, presenting a real challenge for large-scale integrative analyses of multi-center data. Fortunately, converting the absolute abundance to a ratio-based metabolomics profiling using common reference materials enables reliable data integration.

### Ratio-based metabolomics profiling improves quantitative data integration in long-term measurement

**Fig. 5:**
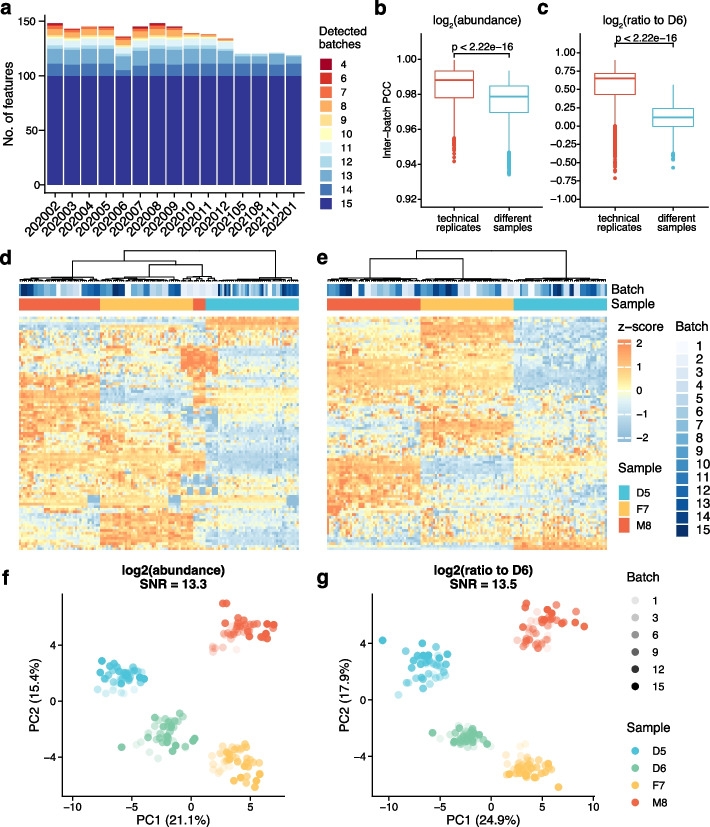
Ratio-based metabolomics profiling improves quantitative data integration in long-term monitoring. **a** Qualitative concordance of metabolite identification. The numbers of metabolites detected in each batch of metabolomic datasets were shown. **b**, **c** Pearson correlation coefficients (PCCs) of pairs of technical replicates and of different Quartet samples were compared using quantitative profiles at the absolute abundance level (**b**) or ratio to D6 level (**c**). **d**, **e** Cross-batch data integration was visualized by hierarchical cluster analysis (HCA) at absolute abundance level (**d**) and ratio to D6 level (**e**). **e**, **f** Cross-batch data integration assessment using signal-to-noise ratio (SNR) by principal component analysis (PCA) at absolute abundance level (**f**) and ratio to D6 level (**g**)

In order to evaluate the long-term stability of metabolomics profiling, we generated a total of 15 batches of Quartet metabolomic data in T-L4 over a period of 2 years. T-L4 metabolomics profiling uses a standard calibration curve to quantify the concentrations of each metabolite, which is regarded as one of the more reliable quantification approaches. We first evaluated the qualitative concordance of detected metabolites in long-term measurements. We found that only 100 out of 148 metabolites (67.6%) were detected in all 15 batches of datasets (Fig. 5a). Thus, the stability of metabolite identification and reporting was still not ideal, even for the absolute quantification metabolomics strategy.

To further evaluate the quantitative stability of long-term metabolomic measurement at the absolute concentration level, we first compared the differences between PCCs of technical replicates and those between different reference sample groups. There were significant differences between the two types of PCCs (Fig. 5b). HCA and PCA were also used to visualize the magnitude of technical variation in data integration at the absolute concentration level. Most of the samples in the integrated long-term metabolomic dataset were clustered into different sample groups, but several M8 samples misclustered into the F7 sample group (Fig. 5d, f). In addition, the Quartet signal-to-noise ratio was calculated to be 13.3 for the integrated dataset, indicating a good separation among different Quartet samples.

After converting the absolute concentration values to a ratio scale relative to those of the same reference material (D6) on a metabolite-by-metabolite basis per batch, the difference between PCCs of technical replicates and PCCs of different samples increased dramatically from 0.009 (Fig. 5b) to 0.532 (Fig. 5c). Similar results were observed by HCA and PCA plots (Fig. 5e and g). After the ratio-based scaling, all the samples clustered correctly into known Quartet sample groups (Fig. 5e). The Quartet signal-to-noise ratio was improved slightly from 13.3 to 13.5 (Fig. 5g). Using the Levey-Jennings plot, we continuously monitored each metabolite measurement across runs (Additional file 1: Fig. S3). There were 57, 14, and 10 metabolites that deviated from the mean beyond ± 3 SD for 3 RMs (D5, F7, and M8), demonstrating evidence of systematic errors (Additional file 1: Fig. S3, up). After ratio-based scaling to D6 sample, the number of systematically deviated metabolites decreased to 8, 11, and 15 for the three sample pairs (D5/D6, F7/D6, and M8/D6), respectively (Additional file 1: Fig. S3, down). These results showed that the reliability of long-term metabolomic measurement can also be improved by converting the absolute concentration values to a ratio scale using common reference materials.

### Construction of ratio-based Quartet metabolite reference datasets for accuracy assessment

**Fig. 6:**
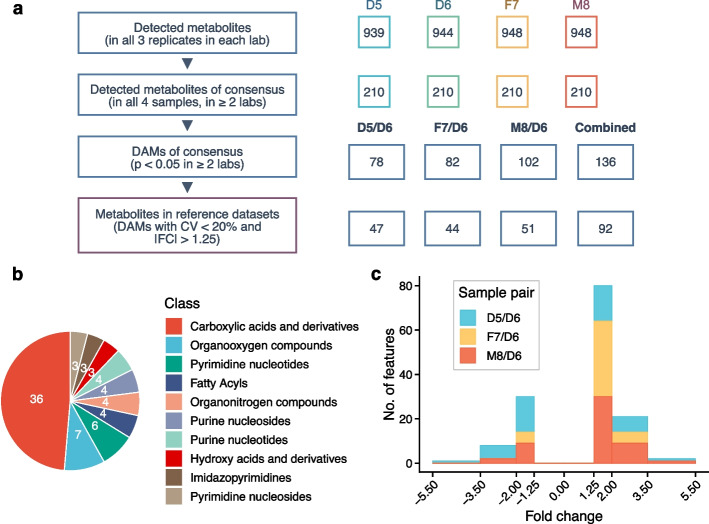
Construction of ratio-based Quartet metabolite reference datasets. **a** The workflow of integration of ratio-based metabolite reference datasets. **b** The number of metabolites in high-confidence reference datasets annotated into 10 classes according to the HMDB database. **c** The distribution histogram of fold changes of the high-confidence reference metabolites for three sample pairs

In order to provide “ground truth” reference datasets for evaluating the accuracy of metabolomic quantification, we constructed ratio-based metabolomic reference datasets for three sample pairs (D5/D6, F7/D6, and M8/D6). The consensus integration process using seven metabolomic datasets from various laboratories is shown in Fig. 6a. The metabolites detected in all three replicates in each dataset were defined as detected metabolites. Next, the union of the detected metabolites in all the seven datasets was 939, 944, 948, and 948 for the four reference materials (D5, D6, F7, and M8, respectively). Secondly, 210 reproducibly detectable metabolites were retained in all four reference samples in more than one dataset. Thirdly, ratio-based values for each sample pair (D5/D6, F7/D6, or M8/D6) were determined using DAMs with *p* < 0.05 in more than one dataset. Finally, the geometric mean of fold changes estimated from each replicate of more than one dataset was defined as the high-confidence ratio-based reference value for each metabolite. Each sample pair (D5/D6, F7/D6, and M8/D6) had 47, 44, and 51 high-confidence metabolites in the first release of the Quartet ratio-based metabolomic datasets (v.1.0) after these steps (Additional file 2: Tables S3-S5), respectively.

According to the HMDB database (https://hmdb.ca), the union of high-confidence reference metabolites (92) for the three sample pairs was categorized into 10 classes (Fig. 6b). With 36 metabolites (39.1%) included in the reference datasets, carboxylic acids and derivatives were the most abundant class. The ratio-based reference values for the high-confidence metabolites for each sample pair were summarized in Fig. 6c, covering a wide range of log2 fold-changes from − 4.4 to 5.1. With the advance in metabolomic technologies and the generation of additional datasets, the Quartet metabolomic reference datasets will be updated periodically through the Quartet Data Portal [43] (http://chinese-quartet.org).

### Best practice for inter-laboratory proficiency test of metabolomics profiling using Quartet metabolite reference materials

**Fig. 7:**
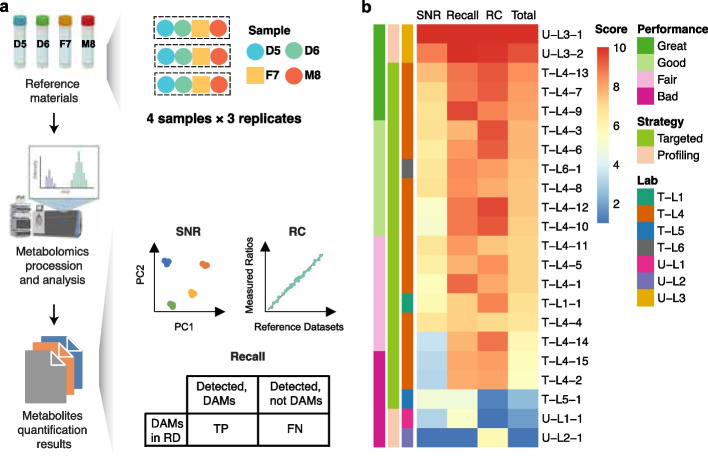
Best practice for inter-laboratory proficiency test of metabolomics profiling using Quartet metabolite reference materials. **a** Flowchart of an inter-laboratory proficiency test using the Quartet metabolite reference materials. **b** Inter-laboratory proficiency for each of the 22 batches of metabolomic datasets using targeted or untargeted strategies with SNR, RC, and recall. The overall performance was classified into four levels (Great, Good, Fair, and Bad)

Inter-laboratory proficiency testing is essential to improving comparability and achieving reliable metabolomics profiling. We recommend profiling the Quartet reference materials for method validation. We provided two types of QC metrics for the quality assessment of quantitative metabolomic datasets. One is the Quartet multi-sample-based SNR, which measures the ability to discriminate the intrinsic biological differences among different reference samples. The other is the quantitative concordance between the query datasets and the reference datasets, calculated by relative correlation (RC). The recall of detected DAMs was recommended to qualitatively assess the sensitivity against the Quartet reference datasets (Fig. 7a).

We evaluated the laboratory proficiency for each of the 22 batches of metabolomic datasets using targeted or untargeted strategies with SNR, RC, and recall. As shown in Fig. 7b, the relative quality rankings using different QC metrics were not concordant. The correlations among the three metric values were relatively low (Additional file 1: Fig. S4). Therefore, a total score would be better to rank the quality of metabolomic datasets. By calculating a total score based on SNR, RC, and recall, with each metric scaled from 0 to 10, we found that the inter-laboratory proficiency is independent of the metabolomic strategy. The top laboratory proficiency was achieved in U-L3 using an untargeted strategy. The three datasets with the worst laboratory proficiency (U-L1, T-L5, and U-L2) were generated in three laboratories using either untargeted or targeted strategies. These results supported the notion that the Quartet metabolite reference materials and related QC metrics were suitable for a wide range of metabolomic technologies using both targeted and untargeted strategies. In addition, the standardized QC workflow for inter-laboratory comparisons can be performed through the Quartet Data Portal [43], where the relative quality ranking among the cumulative metabolomic datasets can be obtained.

## Discussion

As part of the Quartet project [41], we provide the community with the first suite of renewable metabolite RMs with matched DNA [44], RNA [42], and protein [45] isolated from the same immortalized cell lines. The intended use of the Quartet metabolite RMs includes intra-laboratory quality control, inter-laboratory proficiency tests, and quality assurance of large-scale metabolomic data integration. The metabolite RMs were formulated as dried cellular extracts and are suitable for evaluating the performances of analytical steps including chromatography separation, mass spectrometry detection, and data processing. However, they are not suitable for assessing the performances of pre-analytical steps such as cell extraction.

Ratio-based reference datasets for sample pairs (D5/D6, F7/D6, and M8/D6) were defined for assessing the quantification accuracy of various metabolomic technologies. Because the instrumental output can change systematically as a result of various laboratory-developed methods [41, 46], ratio-based quantification provides definite advantages in reducing systematic technical variations and improving data comparability. Therefore, the Quartet ratio-based reference datasets are suitable for proficiency testing of a wide range of targeted and non-targeted metabolomics profiling approaches. Although v1.0 of the metabolite reference datasets covers only 92 metabolites, the reference datasets will be updated periodically through community participation in and contributions to the Quartet Data Portal (http://chinese-quartet.org/) [43]. Our study suggested a paradigm shift from “absolute” to “ratio”-based reference datasets of “ground truth” for calibrating and validating metabolomics profiling.

Another important aspect of the Quartet reference material suite is the intrinsic reference-free QC metrics for objective quality assessment, which is particularly important for various laboratory-developed metabolomic measurements. The Quartet multi-sample-based SNR is defined as the ability to identify inherent differences (biological signals) among the four individual sample groups (D5-D6-F7-M8). Compared to the metrics previously widely used for one-sample-based technical reproducibility in quantitative profiling, the unique Quartet SNR offers higher resolution in identifying serious quality issues. Any QC metrics based on the reference dataset have a clear disadvantage because their assessment is restricted to the limited number of metabolites that are easy to be detected by multiple laboratories. On the other hand, the Quartet-based SNR can be used to evaluate the reliability of global metabolomics profiling without any reference datasets, making it a complementary QC metric for proficiency tests. Using the Quartet metabolite RMs and QC metrics, significant variations across laboratories in the reliability of the metabolomics profiling were observed. We found that the best and worst performing metabolomic datasets were all generated using untargeted metabolomics strategies. This result suggested that intrinsic laboratory proficiency, not instruments or protocols, was most important for reliable metabolomics profiling, consistent with a previous report [47].

More importantly, the Quartet-based SNR can also be used for quality assessment of data integration. To achieve reliable data integration from long-term and cross-laboratory large-scale metabolomics profiling, we recommend using common reference materials per-batch along with study samples. As long as the integrated datasets maintain the ability to differentiate the different Quartet samples, the reliability of the metabolomic data from the study samples for further exploratory metabolomic biomarker discovery is assured. Our study also demonstrated that ratio-based profiling, by scaling the absolute abundance of study samples (such as D5, F7, and M8) relative to those of a concurrently measured common reference sample (such as D6) on a metabolite-by-metabolite basis, will empower large-scale data integration. The ratio-based metabolomics profiling was suitable for internal quality control in longitudinal measurement within a laboratory, and it can also be used to calibrate the metabolomics profiling in multiple centers. Even if the metabolomic methods were developed using different wet-lab operation procedures on different LC–MS instruments, the ratio-based metabolomic data integration was reliable enough for differentiating the various Quartet reference materials. The intrinsic batch-effect resistant characteristics of ratio-based profiling are also demonstrated by other quantitative omics profiling technologies, such as methylomics, transcriptomics, and proteomics [41, 42, 46].

There are some limitations beyond the scope of this study. First, the Quartet metabolite reference materials were extracted cellular metabolites in the form of lyophilized power and could not be applied to the QC of the sample preparation procedures. Moreover, the metabolites extracted from cells could not fully cover metabolites from other sources of biospecimen, such as plasma, serum, and tumor tissues, which may hinder the wider application of the Quartet reference materials, especially when the matrix of the study samples is largely different from cellular extracts.

## Conclusion

In summary, the present study provides the community with metabolomic reference materials, reference datasets, and the corresponding quality metrics for proficiency testing of a wide range of metabolomic technologies. Additionally, ratio-based metabolomics profiling using common reference materials will improve cross-laboratory comparability and long-term data stability, ensuring large-scale metabolomic data integration. Overall, our study provides a new paradigm for developing metabolomic reference material suites for proficiency tests, ensuring the reliability of large-scale metabolomic studies.

## Methods

### Cell culture

Quartet immortalized B lymphoblastoid cell lines were established through infection with Epstein-Barr virus (EBV) [48] and culturing using the protocols described in the Quartet main paper [41]. Lymphoblastoid cell lines were cultured in RPMI 1640 supplemented with 15% non-inactivated FBS and 1% penicillin–streptomycin. Flasks were incubated in a horizontal position at 37 °C under 5% CO_2_. Cell cultures were split every 3 days for maintenance, as described in the literature [49]. Cells growing in suspension were centrifuged at 300 g for 5 min to obtain cell pellets, washed twice with cold PBS, and store at – 80 °C. About 10^9^ cells per cell line were collected for preparing Quartet metabolite reference materials. The cell lines have been authenticated by STR profile, karyotype, PCR mycoplasma, and sterility testing. There was no mycoplasma contamination found.

### Preparation of Quartet metabolite reference materials

Metabolites were extracted from EBV immortalized lymphoblastoid cell lines at the Human Metabolomics Institute, Inc. (Guangdong, China). First, we thawed cells (11 tubes per sample, 10^8^ cells per tube) slowly on an ice bath to minimize potential sample degradation, and then added 2.4 mL of ice-cold methanol solution (methanol to water = 6:1) to each tube of samples. Then, the ice water bath was under ultrasonic treatment for 3 times, each time for 3 s, with an interval of 2 min. After the cell mass at the bottom of the tube was completely broken, the cell extracts were centrifuged at 4500 g for 20 min (Allegra X-15R, Beckman Coulter, Inc., Indianapolis, IN, USA), and then the supernatant was transferred to a new centrifuge tube. Eleven external controls were spiked into the supernatant at known concentrations as internal standards (Additional file 2: Table S1).

For each Quartet sample, the supernatant containing metabolites extracted from 10^9^ cells was aliquoted into 1000 vials using an automated liquid handler (Biomek 4000, Beckman Coulter, Inc., Brea, California, USA). After centrifugation at 4 °C under vacuum (Labconco, Kansas City, Missouri, USA) for 50 min, the Quartet metabolite reference materials were obtained in the form of lyophilized power. We stored the Quartet metabolite reference materials at – 80 °C for long-term use.

### Data generation

We distributed 12 vials (triplicates for each Quartet sample) of the Quartet metabolite reference materials as a batch to each laboratory for data generation. Each batch of samples was run in the same order across all laboratories (D5-1, D6-1, F7-1, M8-1, D5-2, D6-2, F7-2, M8-2, D5-3, D6-3, F7-3, and M8-3).

Metabolomic data were generated in each laboratory using different HPLC/UPLC or MS/MS platforms and experimental protocols (details in Additional file 2: Table S2).

#### T-L1/U-L1

Reference materials were first centrifuged before adding 200 μL acetonitrile–water (1:1, v/v) to reconstitute the samples. The solution was then centrifuged at 14,000 rcf for 15 min at 4 °C to obtain the supernatant for LC–MS analysis.

#### U-L2

One hundred microliters of 50% acetonitrile was added to reconstitute (containing an isotope-labeled internal standard mixture) the reference materials. The solution was vortexed for 30 s and sonicated in an ice-water bath for 10 min. After centrifugation at 13,000 rpm for 15 min at 4 °C, the supernatant was used for LC–MS analysis.

#### U-L3

Five hundred microliters of an ice-cold 80% methanol solution was added to dissolve the reference materials. Then, the solution was divided into five fractions for LC–MS analysis using four different methods, including two separate reverse phases (RP) UPLC-MS/MS with positive ion mode electrospray ionization (ESI), a RP UPLC-MS/MS with negative ion mode ESI, and a HILIC UPLC-MS/MS with negative ion mode ESI. Samples were placed briefly on a TurboVap® (Zymark) to remove the organic solvent and stored overnight under nitrogen before analysis.

#### T-L4

Three hundred fifty microliters of an ice-cold 50% methanol solution was added to resolve the reference materials. The samples were then stored at 20 °C for 20 min and then centrifuged at 4000 g for 30 min at 4 °C. One hundred thirty-five microliters of supernatant was transferred to a 96-well plate, which contained 15 μL of internal standard per well. Serial dilutions of standard samples were added to the same plate. The plate was sealed for LC–MS analysis.

#### T-L5

Five hundred microliters of a 10% methanol solution was added to dissolve the reference materials and then injected into LC–MS for analysis.

#### T-L6

One hundred microliters of reconstituted solution (acetonitrile to water = 1:1) was added to resolve the dried metabolites. After vortexing for 1 min, the solution was centrifuged at 15,000 rpm for 10 min at 4 °C. About 60 μL of the supernatant was transferred for LC–MS analysis.

### Data processing

Raw data acquired using LC–MS were pre-processed by each participating laboratory to provide structured data in.xls format for subsequent statistical analysis. Chromatography-MS data for a single sample are a matrix of m/z versus retention time (or index) versus ion current or intensity.

#### T-L1

MRM raw data were extracted by MRMAnalyzer (R), and the peak area of each metabolite was obtained. More detailed descriptions can be found in reference [50].

#### U-L1

The raw data was converted into mzXML format by ProteoWizard. The researchers used the XCMS program for peak alignment, retention time correction, and peak area extraction. For structure identification of metabolites, accurate mass matching (< 25 ppm) and secondary spectrum matching were used to search the laboratory’s in-house annotation database.

#### U-L2

ProteoWizard software was used to convert the original mass spectrum into mzXML format and XCMS for retention time correction, peak identification, peak extraction, peak integration, and peak alignment. An in-house annotation database was used in parallel to identify the metabolites.

#### U-L3

ThermoFisher Scientific software Xcalibur QuanBrowser was used for peak detection and integration. A detailed description of data processing, including chromatographic alignment, QC practices, and compound identification, can be found in reference [51].

#### T-L4

The raw data files generated by UPLC-MS/MS were processed using the QuanMET software (v2.0, Metabo-Profile, Shanghai, China) to perform peak integration, calibration, and quantitation for each metabolite.

#### T-L5

The detection of samples using MRM (Multiple Reaction Monitoring) was based on the T-L5 in-house annotation database. The Q1, Q3, RT (retention time), DP (declustering potential), and CE (collision energy) were used for metabolite identification. The data files generated by HPLC–MS/MS were processed using SCIEX OS Version 1.4 to integrate and correct the peak. The area of each peak represents the relative content of the corresponding metabolite.

#### T-L6

The MRM raw data were extracted by OS-MQ software (AB SCIEX), and the peak area value of each metabolite was obtained.

### Data integration

We collected 264 metabolomic profiles at the quantification level, with HMDB (Human Metabolome Database, https://hmdb.ca) IDs for the metabolites provided by each laboratory.

We integrated these metabolomic profiles first by HMDB IDs and then by metabolite names. Metabolites were annotated into different classes according to HMDB (https://hmdb.ca/system/downloads/current/hmdb_metabolites.zip, released on 2021–11-17).

### Performance metrics

Based on Quartet metabolite RMs, we constructed three types of performance metrics to comprehensively evaluate the performance of metabolomics profiling in each laboratory.

### Signal-to-noise ratio (SNR)

We measured SNR by comparing the average Euclidean distances between different Quartet samples (“signals”) to those between different technical replicates of the same Quartet sample (“noises”) computed based on the first two principal components of PCA. SNR was defined as the following equation:

$$SNR=10\times {log}_{10}(\frac{m\times \left(\genfrac{}{}{0pt}{}{n}{2}\right)}{\left(\genfrac{}{}{0pt}{}{m}{2}\right)\times n\times n}\times \frac{\sum_{x=1}^{m}\sum_{y=1}^{m-x}\sum_{i=1}^{n}\sum_{j=1}^{n}\sum_{p=1}^{2}{W}_{p}(P{C}_{p,i,x}-P{C}_{p,j,y}{)}^{2}}{\sum_{x=1}^{m}\sum_{i=1}^{n}\sum_{j=1}^{n-x}\sum_{p=1}^{2}{W}_{p}(P{C}_{p,i,x}-P{C}_{p,j,x}{)}^{2}})$$

Here, $m$ is the number of different groups of samples and $n$ is the number of technical replicates of the same sample group. The variances explained by the *p*th principal component ($P{C}_{p}$) were noted as ${W}_{p}$.$P{C}_{p,i,x}$,$P{C}_{p,j,x}$, and $P{C}_{p,j,y}$ represent the value of *i*th and *j*th replicates of sample $x$ or sample $y$ on *p*th principal component, respectively.

### Recall

We computed recall for the assessment of qualitative agreement with the reference datasets. Here, recall is the number of measured DAMs (*p* < 0.05, *t* test) divided by the number of DAMs that should be identified in reference datasets.

### Relative correlation (RC)

We measured RC to assess quantitative consistency with the reference datasets. First, we calculated the average log2 abundance of each metabolite in each Quartet sample. Based on the average log2 abundance, we computed the relative abundance values of metabolites in each sample pair (log2 ratios to D6) that overlapped with the reference datasets in each laboratory. Finally, the Pearson correlation was computed between the measured relative abundance values and the reference datasets.

### Statistical analysis

We used R version 4.0.5 and associated packages to perform all statistical analyses. All statistical tests described in this work were two-sided. Tests involving comparisons of distributions were done using “wilcox.test” unless otherwise specified. The intraclass correlation coefficient (ICC) was computed based on package *irr* (v0.84.1), using a two-way model and estimated by the agreement between raters to compute differences in judges’ mean ratings. We plotted all results based on the R packages *ggplot2* (v3.3.6), *cowplot* (v1.1.1), *ComplexUpset* (v1.3.3), *ggpubr* (v0.4.0), *ggsci* (v2.9), and *GGally* (v2.1.2).

### Supplementary Information

**Additional file 1:** **Fig S1.** | Preparation of Quartet metabolite reference materials. **Fig S2.** | Concordance of detected metabolites among laboratories. **Fig S3.** | Ratio-based metabolite profiling improves the stability of continuous monitoring of each metabolite measurement. **Fig S4.** | Scatter plot matrices for SNR, Recall and RC.

**Additional file 2:** **Table S1.** External controls spiked in the Quartet metabolite reference materials. **Table S2.** Experimental methods of metabolomics profiling in each datasets. **Table S3.** Reference datasets of D5/D6. **Table S4.** Reference datasets of F7/D6. **Table S5.** Reference datasets of M8/D6.

**Additional file 3.** Review history.

## Data Availability

Materials availability The Quartet metabolite reference materials can be requested for research use from the Quartet Data Portal (http://chinese-quartet.org/) under the Administrative Regulations of the People’s Republic of China on Human Genetic Resources. Data and code availability The datasets generated and analyzed during the current study and the Quartet metabolite reference datasets are available in the Synapse repository, 10.7303/syn53190805 [52]. The source codes for the data analyses are available under the terms of the Eclipse Public License 2.0 and GNU General Public License at https://github.com/chinese-quartet/quartet-metqc-report and 10.5281/zenodo.10427770 [53, 54].
