# Supplementary material for: Quartet metabolite reference materials for inter-laboratory proficiency test and data integration of metabolomics profiling
Source: Genome Biol. 2024 Jan 24;25:34. doi: 10.1186/s13059-024-03168-z (PMC10809448; doi:10.1186/s13059-024-03168-z)
Supplement: Supplementary file 1 — Additional file 1: Fig S1. | Preparation of Quartet metabolite reference materials. Fig S2. | Concordance of detected metabolites among laboratories. Fig S3. | Ratio-based metabolite profiling improves the stability of continuous monitoring of each metabolite measurement. Fig S4. | Scatter plot matrices for SNR, Recall and RC. [file 13059_2024_3168_MOESM1_ESM.docx]

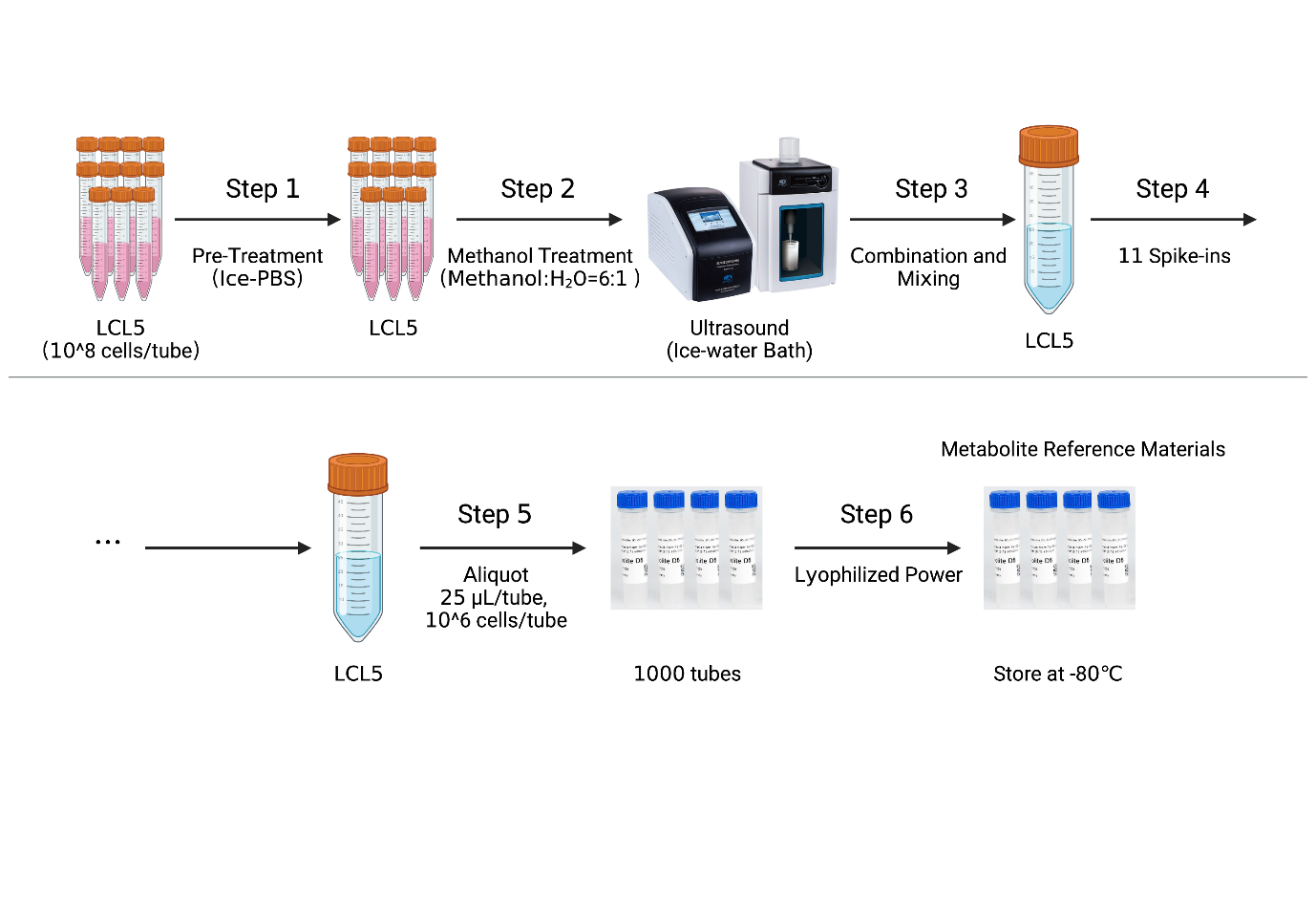


### Fig. S1 | Preparation of Quartet metabolite reference materials.

### Fig. S2 | Concordance of detected metabolites among laboratories.

The intersection size of detected metabolites among seven datasets generated in different laboratories was shown.


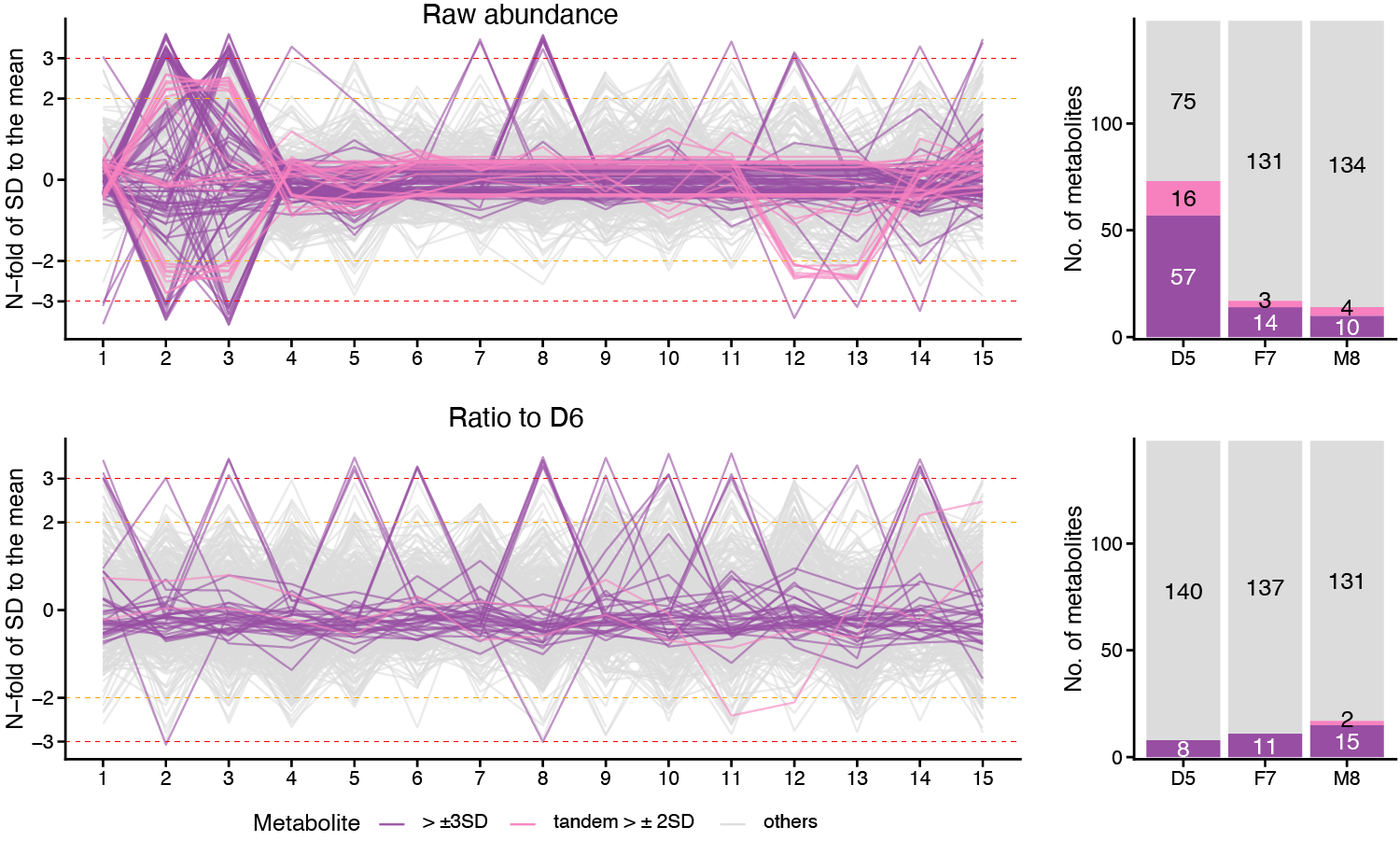


### Fig. S3 | Ratio-based metabolite profiling improves the stability of continuous monitoring of each metabolite measurement.

The Levey-Jennings plot of metabolites detected in all 15 batches was shown. Different colors represent different groups of metabolites, indicating absolute difference to the mean were larger than 3 SD (> ±3SD), or tandemly larger than 2 SD (> ±2SD), and others.

### Fig. S4 | Scatter plot matrices for SNR, Recall and RC.
