# Supplementary material for: Quartet metabolite reference materials for inter-laboratory proficiency test and data integration of metabolomics profiling
Source: Genome Biol. 2024 Jan 24;25:34. doi: 10.1186/s13059-024-03168-z (PMC10809448; doi:10.1186/s13059-024-03168-z)
Supplement: Supplementary file 3 — Additional file 3. Review history. [file 13059_2024_3168_MOESM3_ESM.docx]

Review History

**First round of review**

**Reviewer 1**

**Were you able to assess all statistics in the manuscript, including the appropriateness of statistical tests used?**

No, I do not feel adequately qualified to assess the statistics.

**Comments to author:**

Zhang et al examined data integration among different laboratories and different platforms. Much of the information is very useful to the readers. For example, many metabolomists believe that the Untargeted method is superior for comprehensive analysis, but the authors' results show that it is not much different from the Targeted method. Most DAMs were also found to be more highly expressed in D6 than in D5, F7, or M8 in T-L5. However, in U-L3, most DAMs were more highly expressed in D5, F7, and M8 than in D6. This means that, as Zhang et al pointed out, high repeatability does not necessarily translate into high resolution to discriminate biological differences among different sample groups. It is also interesting to note that filtering and improving data quality using criteria of CV<30% and ICC>0.4 did not change the relative quality ranking of the batches and did not result in a match in data ranking among laboratories. The authors propose a practical solution to the batch effect in absolute comparisons and suggest that it is better to use relative comparisons.

Of concern is that many of the metabolites detected were reported by only one laboratory, and only six metabolites were reported in all seven metabolome data sets. This raises the possibility that the target substances being quantified and compared are misidentified. It is not surprising that the quantification values would be different if there were different substances to be compared. The authors should comment on this point.

**Reviewer 2**

**Were you able to assess all statistics in the manuscript, including the appropriateness of statistical tests used?**

Yes, and I have assessed the statistics in my report.

**Comments to author:**

This manuscript aims for the development of a new set of cell lines reference materials (RM) and a thorough characterisation of the metabolomic analysis of the RM, mostly based on an inter-laboratory ring study. The experiments are well described and the statistical methods are adequate and even innovative, to a certain extent. Overall, this is an interesting exercise of inter-laboratory cross-calibration but I have serious doubts that the overall results warrants the merit required for its publication in this high profile Journal. Specifically, the authors detected a lot of inter-variability within the measurements, as expected since the protocols from the different laboratories are quite different. This is a well-known limitation in metabolomics and the study seems a bit naive in this regard. Yet, the authors made an important effort to extract the "ground truth" (in their own terms) by applying some statistical metrics to the data obtained from the different laboratories. This is interesting (for instance the definition of SNR) but it has been insufficiently validated: there is no independent cohort for validation of the metrics nor external laboratories that would reproduce the expected metrics. Moreover, the protocols are not totally independent since all the samples were extracted at L4. Finally, it is confusing (and annoying) that all the data has been already deposited in up to five preprints. Is the authors's intention to publish up to five papers? If so, it is not clear to me how to dissect the work.

In summary, authors presented an interesting and amiable effort for standardisation and characterisation of cell extracts for future applications in metabolomics and other -omic techniques. Yet, I find this work too technical and premature for Genome Biology and recommend its publication in a more specialised Journal.

**Authors Response**

**Point-by-point responses to the reviewers’ comments:**

Reviewer #1:


Zhang et al examined data integration among different laboratories and different platforms. Much of the information is very useful to the readers. For example, many metabolomists believe that the Untargeted method is superior for comprehensive analysis, but the authors' results show that it is not much different from the Targeted method. Most DAMs were also found to be more highly expressed in D6 than in D5, F7, or M8 in T-L5. However, in U-L3, most DAMs were more highly expressed in D5, F7, and M8 than in D6. This means that, as Zhang et al pointed out, high repeatability does not necessarily translate into high resolution to discriminate biological differences among different sample groups. It is also interesting to note that filtering and improving data quality using criteria of CV<30% and ICC>0.4 did not change the relative quality ranking of the batches and did not result in a match in data ranking among laboratories. The authors propose a practical solution to the batch effect in absolute comparisons and suggest that it is better to use relative comparisons.

Reply:

Thank you very much for your insightful comments. We greatly appreciate your recognition of the value of the Quartet project to the metabolomics community.

Of concern is that many of the metabolites detected were reported by only one laboratory, and only six metabolites were reported in all seven metabolome data sets. This raises the possibility that the target substances being quantified and compared are misidentified. It is not surprising that the quantification values would be different if there were different substances to be compared. The authors should comment on this point.

Reply:

Thank you for your important question. It is truly troublesome and worth mentioning in the manuscript. Indeed, peak misidentification remains one of the major challenges for mass spectrometry based metabolomic profiling (Alseekh Y. et al., Mass spectrometry-based metabolomics: a guide for annotation, quantification and best reporting practices, Nature Methods 2021:18, 747–756), which may contribute partially to the observation that “many of the metabolites detected were reported by only one laboratory, and only six metabolites were reported in all seven metabolome data sets”. The reasons for misidentification include isomers, overlapping compounds that cannot be separated at enough resolution, and in-source degradation products, which can be reduced through in-house manual curation or peak filtering in each laboratory (Alseekh Y. et al. 2021).

Due to the lack of standardized and open source experiments and data processing methods among different laboratories, we admit that we cannot guarantee that all the misidentifications have been removed in each in-house developed metabolomic method. In addition, and more importantly, the reported seven metabolomic datasets were generated using methods that are different during various steps including chromatography separation, mass spectrometry detection, data processing, and metabolite annotation, and each step has its own impact on the metabolomic results. Therefore, biases of selectively detected metabolites were expected, as indicated by the varying numbers of metabolites (ranged from 79 to 462) and hence disconcordance in detected metabolites among different laboratories (six metabolites were detected in all seven metabolomic datasets). As suggested by the referee, we added these comments in the revised manuscript (Lines 226-235).

For comparison of quantification values of the concurrent detected metabolites after in-house data processing, our main finding was “the ratio-based quantification values was comparable among different laboratories”. Therefore, we constructed the “ratio-based Quartet reference datasets” for evaluating the accuracy of quantification and proposed a solution for quantitative data integration of metabolomics profiling. We hope that this clarification addresses your concerns, and we think that our findings helped objectively reveal the real-world intra-laboratory data quality and inter-laboratory comparability of metabolite profiling. The Quartet metabolomics reference materials and the datasets being made publicly available can serve as a foundation for the community to further investigate the underlying limitations of the metabolomics technologies and improve them.
 
Reviewer #2:


This manuscript aims for the development of a new set of cell lines reference materials (RM) and a thorough characterisation of the metabolomic analysis of the RM, mostly based on an inter-laboratory ring study. The experiments are well described and the statistical methods are adequate and even innovative, to a certain extent. Overall, this is an interesting exercise of inter-laboratory cross-calibration but I have serious doubts that the overall results warrants the merit required for its publication in this high profile Journal.

Reply:

We greatly appreciate the referee’s recognition of the novelty, adequacy, and contributions of our work. Regarding the referee’s concern on the level of contributions and significances of our work, we would like to point out a few facts to reflect the advances our work brings to the community.

First, we provided the community with the first suite of cell line derived metabolite reference materials and datasets, which can be used for the much-needed intra-lab quality control and cross-lab proficiency testing of a wide range of metabolomics technologies using different protocols and instruments. All the resources were publicly available through the Quartet Data Portal (http://chinese-quartet.org/).

Secondly, we demonstrated that the Quartet based Signal-to-Noise Ratio (SNR) is a key metric for reliability assessment and is not dependent on protocols and technologies. The SNR objectively measures the ability of a metabolomic profiling approach in discriminating the intrinsic biological differences among different sample groups, a key requirement that cannot be assured by technical reproducibility metrics on the basis of one single sample, a common practice widely used by the metabolomics field.

Thirdly, to the best of our knowledge, no prior study has demonstrated the unique advantages of ratio-based profiling for enabling the integration of diverse metabolomics data. We demonstrated that ratio-based metabolomic data are inherently reproducible and batch-effect resistant, hence increasing the chance of discovering reliable biomarkers for realizing precision medicine.

Finally and importantly, the Quartet metabolite reference materials are matched with DNA, RNA, and protein reference materials from the same batch of cell culturing, making them uniquely suitable for quality control and data integration of multiomics research, as we detailed in accompanying manuscripts (Zheng Y. et al., Ratio-based multiomics profiling using universal reference materials empowers data integration, https://doi.org/10.1101/2022.10.24.513612v1).

Accordingly, we extensively revised our manuscript to address the referee’s concerns and highlighted the significances of our study. We sincerely hope that the level of contributions and the significances of our work will be better recognized when it is published in a high profile journal such as Genome Biology.


Specifically, the authors detected a lot of inter-variability within the measurements, as expected since the protocols from the different laboratories are quite different. This is a well-known limitation in metabolomics and the study seems a bit naive in this regard.

Reply:

Thank you very much for the comments. We are well aware of the widely accepted view in the metabolomics field that inter-laboratory variabilities within the measurements are expected since the protocols from the different laboratories are quite different. We note that such an observation and community-wide “consensus” is based on representing metabolomic measurements on an absolute (or single sample) level. However, to the best of our knowledge, no prior study has demonstrated the unique advantages of ratio-based profiling using common reference material(s) for enabling the integration of diverse metabolomics data. We demonstrated that ratio-based metabolomic data are inherently reproducible and batch-effect resistant, irrespective of the protocols or instruments, hence increasing the chance of discovering reliable biomarkers for realizing precision medicine. We feel that there is a need for the field to rethink what metabolomic profiling is exactly measuring and how “results” should be represented.

In addition, to the best of our knowledge, no study has determined which measurements are of good quality in the sense of their relevance to the intended use of the metabolomic technologies. We emphasize that data quality assessments are dependent on the QC metrics being used. In our present study, we designed the Quartet based SNR metric (Fig. 3c) to objectively measure the ability of metabolomics profiling in detecting the intrinsic biological difference between different groups of samples. We note that a major goal of omics studies is to systematically identify the inherent biological differences among different sample groups (e.g. disease subtypes). Therefore, in our humble opinion, the SNR is a key metric to determine data quality. As shown in Fig. 3A, high variabilities in data quality were observed in our study, as indicated by the SNR values ranging from 4.6 to 27.1 among different datasets. The Quartet based SNR showed high resolution in identifying data quality issues than technical reproducibility of one single sample. A good metabolomics measurement should meet the minimum requirement of being able to discriminate biologically different Quartet sample groups (donors) from technical replicates of the same Quartet sample group. In our humble opinion, the comprehensive reference materials and datasets resources along with the multi-sample based SNR metric being provided to the community will facilitate the objective assessment of intra-laboratory and inter-laboratory quality of a wide range of metabolomic technologies.


Yet, the authors made an important effort to extract the "ground truth" (in their own terms) by applying some statistical metrics to the data obtained from the different laboratories. This is interesting (for instance the definition of SNR) but it has been insufficiently validated: there is no independent cohort for validation of the metrics nor external laboratories that would reproduce the expected metrics.

Reply:

We greatly appreciate the referee’s recognition of the importance of the "ground truth" for inter-laboratory proficiency testing of metabolomics profiling. We confirm that such metrics based on the “ground truth” have been validated and full reproducible.

Before addressing the referee’s concerns, we want to clarify our definitions of “ground truth” in the manuscript. The first type of “ground truth” is based on the “intrinsic biological differences” among the four Quartet donors. The SNR (Signal-to-Noise Ratio) metric was used to measure the ability of metabolomic measurement to identify the biological differences among the four individual sample groups (D5-D6-F7-M8) and can serve as the basis for evaluating the overall performance of metabolomic profiling. The second type of “ground truth” refers to the ratio-based high-confidence reference datasets, which is integrated from consensus of cross-laboratory, cross-protocol metabolomics measurements. The reference datasets can be used for assessing the accuracy of ratio-based metabolomics profiling. According to the referee’s suggestions, we validated the two types of “ground truth” using independent datasets.

First, we validated the utility of the SNR metric using multi-laboratory metabolomics datasets generated from a completely independent set of four plasma reference samples. The set of sex-differentiating plasma multiomics reference materials, abbreviated as Plasmix (Supplementary Fig. 1), includes M (mixed plasma from healthy males), F (mixed plasma from healthy females), Y (mixing samples M and F in 3:1 ratio), and X (mixing samples M and F in 1:3 ratio). We first conducted principal variance component analysis (PVCA) to visualize the dominant sources of variability in each batch of the Quartet metabolomics data (Supplementary Fig. 2a). As expected, the values of SNR is positively correlated with the percentage of variance dominated by sample types (Supplementary Fig. 2b). Similar results were observed using independent datasets from Plasmix (Supplementary Figs. 2c and 2d, unpublished data). These results supported the SNR can be used to discriminate the data quality in metabolomics community. We also note that the SNR metric has been demonstrated to be universally applicable to other types of omics data (epigenetics, transcriptomics, and proteomics) beyond metabolomics data, as shown in an accompanying paper under revision for Nature Biotechnology (Zheng Y et al.) and discussed with editors of Genome Biology.

Secondly, we validated the utility of the “ground truth” reference datasets using the same workflow based on independent datasets that were not involved in constructing the current reference datasets. The datasets included in the construction of the new reference datasets consisted of batches 2 to 15 from T-L4 and two batches of data generated from separate extracts from the Quartet cell lines in U-L3. We found that the newly constructed ratio-based reference datasets highly correlated with the original reference datasets (Supplementary Fig. 3). These results demonstrated the reliability of the “ground truth” reference datasets using our consensus integration workflow.

Importantly, we developed the Quartet Data Portal (chinese-quartet.org) to encourage users of the Quartet reference materials to upload their own datasets for quality assessment including the calculation of SNR values, among many other functionalities, further facilitating the validation and reproduction of the performance metrics based on the “ground truth” built in the Quartet study design.

Moreover, the protocols are not totally independent since all the samples were extracted at L4.

Reply:

Thank you for the comment. In order to ensure the homogeneity and stability of the reference materials for long-term usage, we generated the Quartet metabolite reference materials that were formulated as dried cellular extracts by collaborating with lab L4. Specifically, we prepared the cell extracts from a large batch of cell pellets (1 × 109 cells per cell line/donor). Then the cell extracts were divided in to 1000 vials and dried under vacuum to obtain the metabolomics reference materials.

We note that the Quartet metabolite reference materials are not suitable for assessing the performance of pre-analytical steps such as cell extraction. The Quartet metabolite reference materials are intended to evaluate the performances of the actual measurement steps including chromatography separation, MS detection, and data analysis. We have added these comments to our revised manuscript (Lines 129-132).


Finally, it is confusing (and annoying) that all the data has been already deposited in up to five preprints. Is the authors's intention to publish up to five papers? If so, it is not clear to me how to dissect the work.

Reply:

Thank you very much for the comments.

The Quartet Project (chinese-quartet.org) has been a huge undertaking over the past seven years of several dozens of organizations within MAQC China and beyond in pursuit of quality control and data integration of multiomics profiling. The authors have had extensive discussions with editors from Nature Biotechnology and Genome Biology during the preparation and before the submission of the manuscripts from the Quartet Project. Eight manuscripts are currently under review or revision in Nature Biotechnology or Genome Biology. It is our sincere hope that these manuscripts, after peer review, will be published in a concerted and collective manner to help move the multiomics field forward.

This article from the Quartet Project focuses on metabolomics. It provides publicly available resources including metabolite reference materials, the ratio-based high-confidence reference datasets and their corresponding the quality metrics. Moreover, this article provides best practice for intra-laboratory quality control and inter-laboratory data integration of metabolomic profiling.

The main paper provides an overview of the Quartet Project, and propose a ratio-based quantitative profiling approach for multiomics data integration (Zheng Y. et al., Ratio-based multiomics profiling using universal reference materials empowers data integration, https://doi.org/10.1101/2022.10.24.513612v1).

Four accompanying papers detail the establishment of reference materials, reference datasets, and QC methods for each type of omics profiling including DNA (Ren L. et al., Quartet DNA reference materials and datasets for comprehensively evaluating germline variants calling performance, https://doi.org/10.1101/2022.09.28.509844), RNA (Yu Y. et al., Quartet RNA reference materials and ratio-based reference datasets for reliable transcriptomic profiling. https://doi.org/10.1101/2022.09.26.507265), protein (Tian S. et al., Quartet protein reference materials and datasets for multi-platform assessment of label-free proteomics, https://doi.org/10.1101/2022.10.25.513670), and metabolite (the current manuscript, Zhang, N. et al. Quartet metabolite reference materials for assessing inter-laboratory reliability and data integration of metabolomic profiling. https://doi.org/10.1101/2022.11.01.514762).

Haplotype-resolved assemblies of the Quartet DNA reference materials and the variant benchmark were also provided (Jia, P. et al. Haplotype-resolved assemblies and variant benchmark of a Chinese Quartet. https://doi.org/10.1101/2022.09.08.504083).

Another paper is dedicated to comprehensively benchmark batch effect correction methods using the Quartet multiomics data (Yu Y. et al., Correcting batch effects in large-scale multiomics studies using a reference-material-based ratio method, https://doi.org/10.1101/2022.10.19.507549).

We also develop the Quartet Data Portal (chinese-quartet.org) for the community to conveniently access and share the Quartet multiomics resources according to the regulation of the Human Genetic Resources Administration of China (HGRAC) (Yang J. et al., The Quartet Data Portal: integration of community-wide resources for multiomics quality control, https://doi.org/10.1101/2022.09.26.507202).


In summary, authors presented an interesting and amiable effort for standardisation and characterisation of cell extracts for future applications in metabolomics and other -omic techniques. Yet, I find this work too technical and premature for Genome Biology and recommend its publication in a more specialised Journal.

Reply:

We greatly appreciate the referee’s positive assessment on the values of our study. We sincerely hope that the level of contributions and the significances of our work will be better recognized when it is published in a high profile journal such as Genome Biology.

**Second round of review**

**Reviewer 1**

The authors seem to take the reviewers' comments seriously.

**Reviewer 2**

I appreciate the response of the authors to my comments but, in the essence, my opinion remains unchanged since the work is essentially the same and, although I appreciate the effort in the standardisation, I still believe that this is mainly a technical improvement and, therefore, more suitable to a more specialised journal.
